# Non-invasive methods for analysing pig welfare biomarkers

**DOI:** 10.17221/17/2024-VETMED

**Published:** 2024-05-27

**Authors:** Martin Svoboda, Michaela Nemeckova, Denisa Medkova, Luca Sardi, Nikola Hodkovicova

**Affiliations:** ^1^Ruminant and Swine Clinic, Faculty of Veterinary Medicine, University of Veterinary Sciences Brno, Brno, Czech Republic; ^2^Department of Animal Protection and Welfare and Veterinary Public Health, Faculty of Veterinary Hygiene and Ecology, University of Veterinary Sciences Brno, Brno, Czech Republic; ^3^Department of Animal Husbandry, Animal Nutrition and Biochemistry, Faculty of Veterinary Hygiene and Ecology, University of Veterinary Sciences Brno, Brno, Czech Republic; ^4^Department of Veterinary Medical Sciences, University of Bologna, Ozzano Emilia (BO), Italy; ^5^Department of Infectious Diseases and Preventive Medicine, Veterinary Research Institute, Brno, Czech Republic

**Keywords:** glucocorticoids, health, housing conditions, pig breeding, sow

## Abstract

At present, the assessment of pig welfare quality has gained significant importance, prompting the exploration of novel biomarkers for this purpose. Traditionally, these biomarkers have been monitored in the blood; however, blood sampling is considered an invasive procedure. Currently, non-invasive methods for collecting samples are emerging as viable alternatives for assessing these biomarkers. This article aims to present the current knowledge regarding the use of non-invasive methods for analysing pig welfare biomarkers, specifically focusing on the saliva, hair, faeces, and urine as matrices to determine these biomarkers. The saliva analysis encompasses various biomarkers, such as cortisol, alpha-amylase, chromogranin A, the total esterase, oxytocin, acute phase proteins, adenosine deaminase, immunoglobulins and parameters of redox homeostasis. Cortisol, a specific biomarker, can be determined in the hair, urine and faeces, while urine samples allow for the analysis of catecholamines as non-invasive markers of pig welfare.

## INTRODUCTION

Nowadays, the welfare of animals, especially those intended for food production, is gaining increasing relevance. The need for an objective assessment of the level of animal welfare is becoming more important (Barnett et al. 2001; Ceron et al. 2022). For this reason, the current research is focused on identifying suitable biomarkers to assess the level of welfare.

One of the most widely accepted definitions of stress is as follows: “The biological response elicited when an individual perceives a threat to its homeostasis” (Moberg 2000). Stressors are events or situations that can trigger stress, negatively impacting a pig’s health and welfare (Coutellier et al. 2007). Thus, there is an evident relationship between stress and welfare. Whenever stress is present, the quality of the welfare is poor. As a result, stress indicators are currently considered for use as biomarkers of welfare levels in pig breeding (Broom and Fraser 2015). For example, cortisol, the most commonly used biomarker of stress in pigs, increases when pigs are subjected to stressors such as castration, weaning, and mixing with unknown pigs. This increase can be measured, quantified, and used as an indicator of the extent of poor welfare (Prunier et al. 2005; Campbell et al. 2013; Broom and Fraser 2015).

## PHYSIOLOGICAL RESPONSES TO STRESS

Pigs perceive stressors as threats to their homeostasis, triggering a variety of physiological responses to stress. The organism has developed various strategies, including behavioural, physiological, and immunological components, all of which are coordinated by the brain (Martinez-Miro et al. 2016):

1. Sympathetic-adrenal-medullary axis response (SAM): Catecholamines, namely epinephrine and norepinephrine, are released from the adrenal medulla when the sympathetic nervous system is activated by a stress stimulus. Biomarkers representing this system include, for example, alpha-amylase and chromogranin A (Fuentes et al. 2011; Escribano et al. 2013; Martinez-Miro et al. 2016).
2. Hypothalamic-pituitary-adrenal axis response (HPA): As part of the stress response, the hypothalamus releases the corticotropin-releasing factor. This results in the release of the adrenocorticotropic hormone (ACTH) by the anterior pituitary gland. Finally, glucocorticoids represented primarily by cortisol, are released by the adrenal cortex (Moberg 2000; Martinez-Miro et al. 2016).
3. Hypothalamic-pituitary-gonadal axis response (HPG): Stress is generally accompanied by an increase in the HPA activity and a decrease in the HPG activity. An example of a hormone used as a stress biomarker in pigs is dehydroepiandrosterone (DHEA) (Chrousos et al. 1998; Martinez-Miro et al. 2016).

## BIOMARKERS IN ALTERNATIVE BIOLOGICAL MATRICES

Until recently, all biomarkers for evaluating stress in organisms were analysed exclusively from the blood, plasma and serum samples. However, blood collection is highly stressful, not only for the animals themselves, which must be restrained, but for the staff in charge of the sampling especially in case of handling heavy animals (Merlot et al. 2011). The associated stress can cause the plasma cortisol to increase within several minutes, leading to the implausibility of the results (Spencer and Deak 2017). For this reason, alternative options have gradually emerged, employing non-invasive methods to assess the welfare level of animals on farms without causing additional stress.

Analysing welfare biomarkers in the saliva, urine, faeces, milk, and hair represents a non-invasive method associated with minimal pain and stress for individuals (Casal 2016; Everding 2021). Moreover, the absence of the need for blood collection, i.e., invasive methods, contributes to the improvement of the animal’s welfare as each blood sampling is considered as a procedure according to Directive 2010/63/EU and, therefore, requires authorisation.

As already stated, cortisol is the most frequently used biomarker of stress in pigs. Cortisol, along with corticosterone, belongs to the glucocorticoid group, a set of hormones produced in the middle zone (*zona fasciculata*) of the adrenal cortex (Reece 2009). Until recently, cortisol or its metabolites was specifically measured in blood samples; however, it can now be determined in other biological samples, such as the saliva, urine, faeces, and hair. Nevertheless, various biological matrices represent cortisol secretion at different periods, which must be considered in the data evaluation (Cook 2012; Casal et al. 2016; Everding 2021). For example, cortisol is secreted into the blood and saliva already within a few minutes after a stressful event, providing information only about short-term concentration levels in the time period of the previous minutes to hours (Bozovic et al. 2013). In contrast, analysing cortisol in the urine provides information about the previous few hours, while, in the faeces, it even offers insights into the previous few days (Palme et al. 1996; Spencer and Deak 2017; Heimburge 2021).

A disadvantage of the cortisol analysis in these biological materials is the fact that the cortisol concentration can be subject to short-term fluctuations caused by factors such as the circadian rhythm and stress induced by sample collection (Heimburge 2021). Furthermore, the measured values can be influenced by the food intake and the physical load of the animals (Otovic and Hutchinson 2015).

## PIG WELFARE BIOMARKERS IN SALIVA

### Methodology of sampling, processing, and analysis of the samples

From a sampling perspective, it is more accurate to refer to oral fluid collection than pure saliva collection, as the latter can only be obtained through salivary gland puncture, which is not a non-invasive method. Thus, although oral fluids are involved, the term saliva is employed in this review.

Several methods of saliva sampling in pigs have been described in the literature. The use of cotton swabs or buds seems to be the most commonly used method of collection (Ekkel et al. 1996; O’Driscoll et al. 2013; Ott et al. 2014; Nemeckova et al. 2022). Saliva can also be collected using a cotton rope (Irwin et al. 2011; Rey-Salgueiro et al. 2018) or a sponge attached to the pen (Gutierrez et al. 2009a; Soler et al. 2013). However, the animals must first get used to these materials; thus, it is advisable to soak the rope or sponge in substances that are attractive to the pigs, e.g., orange juice. This will increase the interest of the animals in the material and help them get accustomed to chewing it.

Each of the sampling procedures, however, has some advantages and disadvantages. For example, the collection of saliva using a cotton rope suspended in the pen is suitable for group sampling, which does not need to be taken at an exact time. The rope should be 1.3–1.5 cm in diameter, depending on the age group of the pigs sampled (Irwin et al. 2011). However, if taking saliva samples using a sponge, an appropriate natural material that is strong enough to withstand chewing by pigs and is not ingested by the pigs during the sampling process must be chosen.

The rope/sponge is then attached to a metal bar, chain or rope, placed in the pen, and left for the pigs to chew for about 30 min to ensure that it is sufficiently saturated with saliva. A sample is then obtained from the rope/sponge by wringing. In this sampling method, it is necessary to check that all the pigs in the pen have accessed and chewed the sampling material. This method of saliva collection appears to be unsuitable for use in the youngest age groups of pigs with stronger signs of exploratory behaviour. In older categories, the rope or sponge saliva collection is more suitable for gathering information on the health status of the herd rather than for determining the biomarkers of stress. This is because ensuring that the sampling was conducted in all the individuals proves challenging (Gutierrez et al. 2009a).

The usage of cotton swabs is accurate and the most suitable method for obtaining an individual sample at a precise time. A cotton swab wound around a stick of a non-absorbent material, or gripped in a pen, is used to swab the oral cavity or to wipe away saliva already flowing around the oral cavity (Nemeckova et al. 2022). In weaned pigs, the Pavlov reflex effect, or stimulation of saliva production by the sounds of the feeding device, can be used to simplify the saliva sampling.

For each saliva sample, regardless of the collection method, precautions must be taken during collection to avoid any possible contamination of the saliva sample by the feed, milk, materials present in the pen, or mud. Also, the possible dilution of oral fluids immediately after drinking by the pigs must be considered; therefore, the samples should be taken with at least a short time gap. In particular, saliva samples contaminated with blood, faeces, or breast milk from suckling piglets may affect the salivary cortisol levels, as cortisol can also be found in these matrices.

Swabs should be centrifuged immediately after swabbing to obtain a sample; a combination of 14 000 rpm and 3 min was found to be suitable (Nemeckova et al. 2022). However, the storage conditions of the saliva samples for the cortisol analysis differ in the literature sources. Lewis (2006) reported that cortisol is stable in a centrifuged saliva sample even after five days at 4 °C and after 3 months of storage at –20 °C; for long-term storage, a temperature of –80 °C is recommended. Nemeckova et al. (2022) monitored the stability and found that there was no significant decrease in the salivary cortisol in samples stored at room temperature for 36 hours. However, most authors recommend that the saliva samples be refrigerated and analysed as soon as possible after collection. Immunochemical methods, such as enzyme-linked immunosorbent assay (ELISA) and radioimmunoassay (RIA), or chromatography, are most commonly used to determine the cortisol in saliva (Escribano et al. 2012a).

### Cortisol

Salivary cortisol is most frequently used as an indicator of acute stress levels. It is employed to primarily assess non-invasive interventions or interventions in the normal biorhythm of pigs, such as changes in the housing type, regrouping, modifications in the breeding technology, transportation, feed deprivation, feed enrichment, or a change of the animal handler. To assess stress levels following invasive interventions, such as castration, tooth resection, or tail docking, it is preferable to measure the cortisol directly in the blood (Prunier et al. 2005; Sutherland et al. 2012; Backus and McGlone 2018).

Only about 10% of glucocorticoids are present in a free form in the blood, allowing them to easily pass into the saliva. The concentrations of cortisol in the saliva are several times lower than those in the blood, specifically, the concentration of salivary glucocorticoids is only about 7–12% of the concentration in the blood (Murray 2001). Nevertheless, the pattern of cortisol elevation in both the blood and saliva remains the same after ACTH administration (Negrao et al. 2004).

To assess the level of stress induced by a particular situation, it is important to take the right time for sampling into consideration. However, the results of the highest cortisol concentrations as a function of time after the stressful event vary among authors. Cook et al. (1996) demonstrated a maximum salivary cortisol concentration at 5 min after the stress load (fixation with a loop). Coutellier et al. (2007) demonstrated the highest salivary cortisol concentration at 4–5 h after the stress caused by the handling and rearrangement of the animals. Nemeckova et al. (2022) focused on monitoring the highest cortisol concentrations following stress loading, where the stressors included castration, tattooing, and vaccination). During the 120-minute monitoring interval, the optimal time for sampling was identified as 40 min after the stress load.

The salivary cortisol concentration is most commonly used to assess the impact of regrouping, transportation, handling, and new housing. Ott et al. (2014) demonstrated that regrouping led to an increase in salivary cortisol, while feed deprivation had no effect (baseline cortisol concentration – 0.29 ± 0.55 μg/dl). A change in housing type has also been demonstrated as a factor that contributes to increased salivary cortisol in sows (before relocation – 34 100.87 ± 2 458.10 pg/ml, after relocation – 38 103.7 ± 4 624.50 pg/ml and 2 h after relocation – 47 226.80 ± 4 645.30 pg/ml) (Nemeckova et al. 2022). When evaluating stress levels during a typical short (approx. 45 min) commercial transport, it was observed that the salivary cortisol concentrations are higher immediately after unloading. However, they return to baseline quickly after unloading (Soler et al. 2013). Similarly, Jama et al. (2016) showed an elevated concentration of salivary cortisol after a 2-hour transport (before transport 3.9 ± 0.06 ng/ml and after transport 15.2 ± 0.07 ng/ml). Perez et al. (2002), however, found that a 15-minute transport was much more stressful for pigs than a 3-hour transport (cortisolconcentration after 15-minute transport – 88.51 ng/ml and after 3-hour transport – 59.05 ng/ml).

### Alpha-amylase and chromogranin A

Salivary alpha-amylase (sAA) is considered one of the non-invasive biomarkers of acute physical and psychological stress, commonly assessed through changes in its activity and concentration (Fuentes et al. 2011; Contreras-Aguilar et al. 2018). sAA is associated with the SAM activation in pigs and humans (Escribano et al. 2018). The reported methods for determining sAA in saliva samples include measuring its activity spectrophotometrically, assessing its concentration by a fluorometric assay, and conducting isoform evaluation by Western blot (Fuentes et al. 2011; Contreras-Aguilar et al. 2018).

Additionally, salivary chromogranin A (CgA) is regarded as a biomarker of the acute stress response and the activation of the sympatho-adrenomedullary system (SAS) and the HPA axis (Escribano et al. 2014a). It is an acidic, soluble protein that is stored and co-released with catecholamines into the bloodstream (Martinez-Miro et al. 2016). CgA is more stable than catecholamines, which are present in low concentrations and break down rapidly. Additionally, CgA is distributed in the secretory vesicles of the endocrine, neuroendocrine, and neuronal cells (Escribano et al. 2014a). The primary methods of determination include an immunofluorometric assay and Western blot (Escribano et al. 2013; Huang et al. 2017).

In a study by Fuentes et al. (2011), sAA was used as a biomarker of stress levels, determined by a spectrophotometric method. The initial saliva sample was collected before inducing stress. Subsequently, acute stress was induced through immobilisation using a nasal snare or loop for a minimum of one minute. Afterward, samples were taken immediately following the restraint stress, and 30 and 60 min thereafter. This study concluded the sAA assay is a simple, reliable and repeatable method for acute stress evaluation and even stress events that happened more than 60 min before the measurement. The Contreras-Aguilar et al. (2018) study evaluated the activity, concentration and isoforms of sAA in pigs exposed to acute immobilisation stress similar to the study by Fuentes et al. (2011). The results of the activity and concentration did not correlate and the inter-individual variability was high; the three possible isoforms of sAA showed a difference in the individuals as well. The authors, however, regard the isoforms of sAA as potential future biomarkers of stress, much like in human medicine. Lopez-Arjona et al. (2020a) evaluated the use of sAA as a stress marker in transport, and saliva samples were collected using chewing sponges. The activity of sAA increased immediately after transport, and even a 4.4-fold increase was observed four hours after the stress.

Regarding CgA, Escribano et al. (2013) conducted a study involving 30 pigs, with 15 of them undergoing a 3-minute exposure to acute stress through immobilisation using a nose snare. Saliva samples were taken before, and 15 and 30 min following the stress-inducing event. The determination of CgA resulted in the high sensitivity and reliability of the developed immunofluorometric assay, yielding results comparable to the cortisol levels detected in the saliva. In a study by Huang et al. (2017), pigs were subjected to immobilisation with a nasal snare or stress induced by enclosure in a steel cage, and saliva samples were collected up to 30 min after the induced stress. CgA was identified as an ideal biomarker of acute stress exhibiting an immediate increase, i.e., within 10 min during restraint stress, and maintaining a significant elevation 30 min after both stressors were introduced.

In comparison to blood measurements, both the sAA and CgA determination and their use as acute stress biomarkers, are non-invasive, relatively stress-free, and simple methods for evaluating stress in pig medicine. In addition, these methods are considered to be cheaper than the determination of adrenalin and noradrenalin (Ceron et al. 2022). However, it needs to be mentioned that the stability of saliva samples, concerning the activity of sAA, seems to be an issue due to bacterial contamination and high enzyme activity. When Escribano et al. (2018) evaluated the stability of sAA in pig saliva, sAA was stable for < 4 days at 4 °C, < 90 days at –20 °C, and < 360 days at –80 °C. A collection of samples was made using chewing sponges, and the concentration of sAA was determined at different time points and temperatures. Although the exact value for which the samples of pig saliva for this analysis are stable is not available, it follows from these values that it is a more appropriate procedure to freeze the sample at –20 °C at least, if we do not immediately process it in the laboratory. That is a difference in relation to humans, where the samples were stable for 5 to 10 days at room temperature. Also, Escribano et al. (2014a) evaluated the stability of CgA during the saliva sample storage. The samples from pigs were taken using chewing sponges and then stored at 4 °C, –20 °C or –80 °C for up to 1 year. The concentration of CgA was measured by an immunofluorometric assay, and the results showed that the CgA concentrations are stable over 2 days at 4 °C. For long-term storage, the temperatures of –20 °C and –80 °C showed no alterations, the same as the repeated freezing of the samples. However, it was strongly recommended that the analysis should be performed within 180 days after sampling to ensure the greatest possible relevance of the obtained data. Additionally, the CgA levels are not affected by the age, gender, or circadian cycle, which is an advantage because, for example, cortisol and IgA are both affected by these factors (Martinez-Miro et al. 2016).

### Total esterase and its components

The total esterase (TEA) is an enzyme that is considered to be a marker of acute stress; however, a study by Contreras-Aguilar et al. (2019) describes the potential of TEA to be used as an indicator of long-term stress, such as pain, distress, and discomfort levels. The main components of TEA in pigs, including butyrylcholinesterase (BChE), lipase (Lip), and carbonic anhydrase isoenzyme 6 (CA-VI), contribute to the TEA activity and increase in the response to acute stress as well (Escribano et al. 2018). Moreover, the measurement of the BChE activity can be a more sensitive marker (Ceron et al. 2022).

TEA is commonly determined by a spectrophotometric assay, as is BChE and Lip (Tecles et al. 2017). The evaluation of the sample stability for the TEA determination showed labile enzymatic activity and resulted in the suggestion that samples should be analysed within 30 days of storage at –20 °C or –80 °C (Escribano et al. 2018).

A study on pigs, evaluating TEA and BChE as stress markers during transport stress (Lopez-Arjona et al. 2020a), involved collecting saliva samples using chewing sponges before transport, immediately after it, and 4 h later. The activity of both TEA and BChE increased immediately after and 4 h after the stress (i.e., TEA baseline = 151.7 IU l^–1^ increased to 190.6 and 277.8 IU l^–1^ immediately and 4 h after the stress, respectively; the BChE baseline = 100 nmol ml^–1^ min^–1^ remained similar immediately after the stress and increased to 600 nmol ml^–1^ min^–1^ 4 h after the stressor). The study confirmed that both of these can be used as markers of stress situations, such as transport.

The Tecles et al. (2017) study primarily aimed to validate the spectrophotometric assay for the TEA analysis from saliva. Pigs exposed to a 1-minute-long nasal snare or loop immobilisation were sampled with chewing sponges before the immobilisation, immediately after it, and 15 min later. The TEA levels were significantly increased immediately after the stress situation and returned to basal values after 15 minutes. Otherwise, the results showed that the TEA assay is repetitive, has high precision and accuracy, and uses a low sample volume. TEA was evaluated as a good marker of acute stress in this study; however, the components of TEA responded differently to acute stress and disease, e.g., lameness.

Regarding the blood measurements of stress, alterations in the activity during the day and dependency on sex should be considered in TEA and its components (Ortin-Bustillo et al. 2022).

### Oxytocin

Hormone oxytocin (OT), important for the maintenance of gestation, labour, and lactation, is raising concern as a marker that increases in relation to positive emotions and social welfare (Ceron et al. 2022). OT is released in response to a stressor and reduces the activity of the HPA axis (Valros et al. 2022).

Samples for OT determinations include serum/plasma, saliva, urine, and cerebrospinal fluid. Saliva is preferred as it can be collected simply and quickly, without additional stress or pain for the animals (Lopez-Arjona et al. 2021). The analyses of OT are based on immunoassays and high-performance liquid chromatography-mass spectrometry analysis (HPLC-MS); however, HPLC-MS has not yet been applied to saliva samples of pigs (Ceron et al. 2022). The study by Lopez-Arjona et al. (2020b) aimed to develop an immunoassay (AlphaLISA) for measuring OT in pig saliva, considering it faster and simpler compared to radioimmunoassays, HPLC-MS, and other ELISA assays.

However, a difference was observed between the developed (monoclonal method that measures free OT vs polyclonal method that measures OT linked to proteins) and commercial immunoassays as these can measure the different forms of OT or OT metabolites in the saliva. Thus, it should be stated in the studies which form of OT was measured and by what assay (Lopez-Arjona et al. 2021).

The study by Lopez-Arjona et al. (2020b), in addition to the development of the previously mentioned AlphaLISA assay, also measured OT levels at different times after farrowing. The study was conducted on saliva collected from healthy sows administered with cloprostenol on day 113 of gestation and oxytocin on postpartum day one. Sampling was conducted on the first day after farrowing, the 9^th^ and 20^th^ day during lactation; all the samples were collected at the time when the sows were suckled. The OT levels were significantly higher at the beginning of lactation (i.e., 1.65 pg ml^–1^), probably due to the physiological occurrences related to suckling and the exogenous administration, and decreased to similar levels on days 9 and 20 (i.e., 1.14 and 1.48 pg ml^–1^, respectively). The study compares these results to a study made by Okrasa et al. (1989) in which the OT levels in the plasma of lactating sows also increased on day 5 and gradually decreased by day 15.

Valros et al. (2022) measured the OT and procalcitonin (a marker of sepsis) levels from pig saliva samples to evaluate the potential of OT as a biomarker of chronic stress, i.e., tail biting. Tail biting is abnormal pig behaviour related to stress and poor welfare, often resulting in lesions, increased stress, or even mortality if severe (Svoboda et al. 2023). The study measured OT using the AlphaLisa method and discovered that the OT levels were lower in a group of pigs with tail lesions than in the control ones assuming that pigs with lesions were under more stress (Valros et al. 2022).

Lopez-Arjona et al. (2020a) exposed female pigs to transport at the end of the fattening period, covering a distance of 15 km to the slaughterhouse. Saliva samples were taken using chewing sponges before transport (baseline = 1 479 pg ml^–1^ for the monoclonal method and 40.8 ng ml^–1^ for the polyclonal method), immediately after it and 4 h later. The OT was measured using the AlphaLisa method, with the following results: the OT concentration decreased significantly at 4 h after the transport (i.e., 1 073 pg ml^–1^ for the monoclonal method and 23.6 ng ml^–1^ for the polyclonal method), probably indicating a decrease in positive feelings and an increase in stress upon arriving at the slaughterhouse. This study concluded that the OT measurement in the saliva can be used as a marker of acute stress. In contrast, the study conducted by Moscovice et al. (2022) did not suggest OT as a reliable marker of emotional valence in pigs. This study measured the OT levels in response to two positive (social play and reunion with group members) and two negative (weaning and brief separation) social challenges in young pigs. Each individual was tested multiple times; however, the salivary OT concentrations did not differ during any of the social interactions. Only during the social isolation did the OT levels decrease non-significantly. We assume the OT analysis from the saliva samples is dependent on the stress level; in these studies, transportation was probably a more stressful event in comparison to any social challenges.

### Stress caused by immune disorders – Inflammatory and infectious markers

By stress, we refer to a range of adverse short-term or long-term effects on the organism that significantly affect its overall reactions, leading to a stress syndrome. One example could be a prolonged subclinical disease that remains untreated due to a lack of clinical signs. Inflammations and infections are quite common in commercial pig production, posing a risk of the hidden and sub-clinical progression of diseases, disease transmission between individuals, and potential economic losses due to reduced performance (Sali et al. 2021). Thus, determining markers of inflammation and infection in saliva provides the advantage of managing health disruptions before disease outbreaks occur.

As examples of these markers, our review discusses acute phase proteins (APPs), adenosine deaminase (ADA), and immunoglobulins (Igs). Additionally, Sali et al. (2021) suggest evaluating multiple markers to observe their dynamics throughout the course of the stress or disease.

#### ACUTE PHASE PROTEINS

Acute phase proteins (APPs) are innate immune markers synthesised in the liver that increase early in response to inflammation, often before clinical signs appear (Ceron et al. 2022). They have the potential to serve as markers of both physical and psychological stress (Martinez-Miro et al. 2016). In response to stress, the SAM axis and HPA axis are activated, leading to the release of catecholamines and glucocorticoids. This increases the release of the proinflammatory cytokine release, such as proinflammatory cytokine interleukin 6 and its response to inflammation. The release of this cytokine leads to the induction of APP production in the liver (Martinez-Miro et al. 2016; Saco and Bassols 2023). Thus, stress is closely related to inflammation, especially when chronic; however, it becomes problematic to evaluate whether only the stress factors or the immune disruption also affected the concentration of APPs.

The primary representatives of APPs include the C-reactive protein (CRP) as a major APP and haptoglobin (Hp) as a moderate APP. Salivary Hp is considered to be a more sensitive marker of some diseases compared to serum Hp (Gutierrez et al. 2009b). However, according to Escribano et al. (2014b), the levels of CRP and Hp in the saliva of healthy pigs are below 10 μg l^–1^ and 1 mg l^–1^, respectively. In cases of inflammation, these values increase to 100 μg l^–1^ and 3–4 mg l^–1^ for CRP and Hp, respectively. When comparing the determination of APPs in the saliva and blood, the concentrations in the saliva are approximately 1 000-fold lower than in the serum (Ceron et al. 2022). Additionally, this poses a challenge in developing assays to measure APPs in saliva, as the methods must be highly sensitive. Time-resolved fluorometric and AlphaLISA assays are commonly employed for determining APPs (Ceron et al. 2022).

For example, a study conducted by Sanchez et al. (2022) on 107 pigs compared the response and correlation of stress and immune factors in salivary samples. Samples were collected using sponges, and the CRP and Hp levels were determined using an immunofluorometric assay. A moderate association was found between the psychological stress markers and both the innate and adaptive immune markers. The CRP and/or Hp concentrations were increased in the pigs with tail-biting, prolapse, diarrhoea and lameness. However, the main aim of the study was to monitor the behaviour of multiple stress and disease markers, and the study did not conclusively establish a unique connection between increased concentrations of APPs and stress or disease.

Some studies have investigated the impact of transportation on the APP levels in relation to the stress experienced by pigs during transport. Even though these APPs were measured in the serum samples, it seems that the CRP increases in response to short-term transport, while the Hp increases in response to long-term transport (Salamano et al. 2008; Weschenfelder et al. 2012).

#### ADENOSINE DEAMINASE

Another inflammatory marker, adenosine deaminase (ADA), is an enzyme expressed in most tissues and body fluids, including the saliva, that plays a role in purine and pyrimidine metabolism (Contreras-Aguilar et al. 2019). ADA has two isoforms, ADA1 and ADA2. ADA1 is present in lymphoid tissues and plays a role in cell-mediated adaptive immunity, i.e., the differentiation of B and T lymphocytes and the maturation of monocytes to macrophages ADA2 is dominant in the plasma and contributes to the function of the haematopoietic system (Contreras-Aguilar et al. 2019; Ceron et al. 2022).

ADA and its isoenzymes were determined by a spectrophotometric automated assay (Tecles et al. 2018). The total ADA is the sum of the activities, which is measured first. Subsequently, the same sample is measured again for the analysis of ADA2 by adding the inhibitor of ADA1 (erythro-9-2-hydroxy-3-nonyl adenine). Finally, ADA1 is determined as the difference between these two measured values (Ceron et al. 2022).

Salivary ADA has shown the potential to be an indicative systemic inflammatory marker. In a study by Sali et al. (2021), female pigs were subjected to lipopolysaccharide (LPS, i.e., endotoxin-inducing systemic inflammation) intramuscularly, alone or in combination with ketoprofen. Saliva samples were collected using chewing sponges at baseline (i.e., ADA = 118.0 IU l^–1^; Hp = 0.33 μg ml^–1^; cortisol 0.32 ng ml^–1^), 4, 24, 48 and 72 h after injection. ADA, cortisol and Hp were measured in the immunoassays. All the measured parameters increased in the saliva samples after the LPS injection, and significantly after 4 h post-administration. However, when the LPS was combined with ketoprofen, the levels of the observed parameters remained stable during the sampling. Based on the determination of the salivary ADA, Hp, and cortisol levels, this study confirmed that pre-treatment with ketoprofen may alleviate the effects of LPS. Moreover, a positive correlation was observed between ADA and Hp (*r* = 0.86, *P* < 0.05).

The study by Kaiser et al. (2021) aimed to evaluate the inflammatory and stress response to postpartum dysgalactia syndrome (PDS) using saliva and serum samples collected from sows every 24 h starting from 60 h prior to parturition. Sampling was conducted using cotton swabs from the PDS-positive sows and healthy sows; ADA and its isoforms were measured spectrophotometrically. Both the ADA1 and ADA2 activity increased after parturition in all the PDS-positive sows, while no changes were observed in the control sows over time.

During the study by Gutierrez et al. (2017), saliva samples were collected from pigs exhibiting various signs, i.e., inflammation, gastrointestinal and respiratory disorders, and growth retardation. Sampling was conducted using chewing sponges, and the results were compared to saliva samples taken from healthy pigs. ADA, including its isoforms, was measured using an automated spectrophotometric assay. This study also assessed the levels of Hp. The results showed that all the analysed parameters were elevated in the pigs with inflammation, gastrointestinal, and respiratory disorders. However, the growth-retarded pigs lacked inflammatory activation and exhibited similar marker activity levels. Concretely, the baseline for ADA was 140 IU l^–1^, which remained similar in the growth-retarded pigs, i.e., 180 IU l^–1^; however, increased to 426.6 IU l^–1^ in the inflammatory pigs, 536.6 IU l^–1^ if gastrointestinal disorders occurred, and was 440.1 IU l^–1^ in the case of respiratory disorders. The baseline for Hp was 0.77 μg ml^–1^ in the healthy pigs and was 0.45 μg ml^–1^ in the growth-retarded pigs; which increased to 2.0, 2.83 and 1.26 μg ml^–1^ in the case of inflammation, gastrointestinal and respiratory disorder, respectively. As a result, the measurement of ADA proved to be a simple, rapid, and non-invasive method for disease detection. Moreover, the diagnostic value of the ADA activity was higher than that of Hp.

The ADA evaluation showed potential as a marker of chronic stress as well. Contreras-Aguilar et al. (2019) studied the enzymatic activity of ADA and its isoforms in serum and saliva samples from four species: dogs, horses, cows, and pigs. Samples of serum and saliva from both control pigs and pigs with lameness (*n* = 20) were taken in the last phase of fattening, and the ADA levels were determined using a spectrophotometric automated assay. The total ADA activity was much higher in the saliva than in the serum samples, i.e., 100-fold higher. Specifically, both the total ADA and ADA1 increased in the saliva samples of the pigs with lameness, while ADA2 increased in the serum of those pigs. Consequently, ADA was identified as a promising marker of stress factors in pigs, and the saliva as an appropriate organic fluid for measuring its activity.

In the study by Valros et al. (2022), the effects of chronic stress, such as tail biting, were evaluated using multiple saliva biomarkers of stress, as discussed above. ADA, including its isoenzymes, was assessed spectrophotometrically. ADA did not exhibit any significant variation across the groups, but ADA2 positively correlated with the Hp (*r* = 0.52, *P* = 0.005), monoclonal (*r* = 0.47, *P* = 0.01) and polyclonal oxytocin (*r* = 0.58, *P* = 0.001), and cortisol levels (*r* = 0.48, *P* = 0.007) in a group of pigs with tail-biting lesions. Furthermore, Contreras-Aguilar et al. (2019) reported a correlation of the ADA with pain scores in lame and prolapsed pigs.

In conclusion, measuring ADA and its isoforms in the saliva is a promising tool for evaluating chronic stress and inflammatory diseases. However, the study by Ortin-Bustillo et al. (2022) reported an increase in the individual differences and sex-dependency when analysing ADA. Therefore, these factors should be considered.

#### IMMUNOGLOBULINS

Immunoglobulins (Igs) are associated with the immune system, offering crucial insights into an individual’s health status (Escribano et al. 2012b). The salivary increase of immunoglobulins, indicative not only of the adaptive immune system, but also of stress, is particularly considered for IgA, as an observed concentration increase was noted in response to stress (Escribano et al. 2014b).

In saliva, the Ig classes G, M, and A are present, with IgG and IgM delivered from the blood. The levels of IgG and IgM in saliva samples correlate with those detected in the blood. Their concentrations provide information primarily about infectious diseases. In contrast, IgA is produced by the salivary glands, and its levels are more closely related to stress than just infections (Ceron et al. 2022). However, IgA appears to be dependent on the circadian rhythm; with levels being lowest in the morning and evening and rising throughout the day. This aspect must be considered as a factor in experimental studies (Muneta et al. 2010). Another factor that needs to be considered is that the Ig concentrations differ in the saliva of healthy pigs, whereas IgA occurs at a concentration of approx. 100 mg/l, IgM at 20 mg/l and IgG at approx. 10 mg/l (Escribano et al. 2012b).

The method of determination of IgG, IgM, and IgA in saliva samples is based on immunoassays such as ELISA, as validated in porcine saliva samples by Escribano et al. (2012b), who described the method to be simple and minimally invasive for evaluating the hormonal immune status.

The IgA is considered a marker of both acute and chronic stress. In a study by Muneta et al. (2010), male piglets were subjected to acute restraint stress, i.e., immobilisation of pigs with iron fences in the corner of the room. IgA was determined by the ELISA method. Saliva samples were taken before, 10 and 20 min after the start, and 10 min after the finish of the stress. IgA increased 10 min after the start of the stress and remained high after 20 min in saliva; however, the IgA levels returned to the basal level 10 min after the stress stopped. This study also evaluated salivary cortisol, which remained elevated after 10 min post the stressor implication, and the sAA levels, which were comparable to the basal levels throughout the entire trial.

The relationship between IgA and endotoxaemia was reported by Escribano et al. (2014b). LPS was administered to 10 pigs. LPS (a dose of 30 μg/kg that was increased by 12% with each subsequent treatment) was administrated three times at 48 h intervals. The saliva samples were collected 3 days before the first LPS challenge and on the day of each subsequent LPS challenge. IgA was analysed using the ELISA method. The IgA concentration increased 100-fold on each day of the LPS challenge compared to the baseline and IgA was considered as a marker of chronic stress in this study.

### The redox homeostasis and oxidative stress

The evaluation of redox homeostasis is the assessment of the balance between oxidative stress and antioxidant defences. Oxidative stress is defined as an imbalance between reactive oxygen (ROS) and nitrogen species (RNS) and antioxidant protection. The radicals are by-products of various biochemical reactions, the production of which is stimulated by internal and external stimuli, and are highly active and unstable due to the presence of unpaired electrons. The maintenance of redox homeostasis is managed by the antioxidant system in the organism and is crucial for the animal’s overall health, whereas an imbalance can lead to various diseases (Hao et al. 2021; Surai and Earle-Payne 2022).

Ceron et al. (2022) summarised the methods of measuring the total redox homeostasis in saliva into three assays: Trolox equivalent antioxidant capacity (TEAC), cupric reducing antioxidant capacity (CUPRAC), and ferric reducing ability of saliva (FRAS). In addition to these, uric acid and catalase, advanced oxidation protein products (AOPP), ferrous oxidation-xylenol orange (FOX), peroxide activity (POX-Act), and reactive oxygen-derived compounds (d-ROMs) can be measured in the saliva (Ceron et al. 2022; Lopez-Martinez et al. 2022).

The determination of the indicators of redox homeostasis is quantified spectrophotometrically (Ceron et al. 2022). The determined concentrations are usually corrected by the protein concentrations; however, some studies discuss whether it is productive as it may distort the results. In particular, a study in humans where uric acid was measured showed that the correlation between serum and saliva results was affected if the concentrations detected in the saliva were not normalised to the protein concentration (Gonzalez-Hernandez et al. 2019; Lopez-Martinez et al. 2022).

In pig blood, the levels of reactive oxygen species (ROS) were reported to increase in response to transport, weaning, gestation, and lactation (Rubio et al. 2019). However, not many studies have evaluated their concentrations in saliva samples and their correlation to acute or chronic stress.

The pilot study by Lopez-Martinez et al. (2022) evaluated the salivary biomarkers of the redox status in response to sepsis induced by an LPS challenge and non-septic inflammation induced by turpentine oil in pigs (*n* = 15; i.e., 5 pigs per group). The samples were collected using chewing sponges 24 h before, and 3, 6, 24, and 48 h after the application. The redox markers were measured spectrophotometrically. In septic pigs, the levels of POX-Act and d-ROMs increased 3 h after the application, whereas the AOPP increased 24 h after it. The levels returned to the baseline 24 h after the challenges. CUPRAC, FRAS, and TEACT showed higher concentrations after 24 h in the LPS-challenged pigs. The study concluded that experimentally provoked sepsis induces an increase in oxidants that can be measured in the saliva; thus, these can be used as biomarkers.

Rubio et al. (2019) observed the levels of redox markers during the lactation period in sows. A total of 14 clinically healthy sows were studied around farrowing, and saliva samples were taken on days 1, 9, and 20 of lactation using chewing sponges. The oxidant markers were measured spectrophotometrically. All of the analysed markers followed a similar pattern of continuous decrease from day 1 to day 20 of lactation. It was concluded that the biomarkers, i.e., TEAC, CUPRAC, FRAP, AOPP, uric acid, and hydrogen peroxide, can increase in situations of oxidative stress, such as lactation, and that their determination in saliva samples is a promising tool for the evaluation of stress in pigs.

## PIG WELFARE BIOMARKERS IN HAIR

### Methodology of sampling, processing and analysis of samples

As an example of the procedure for taking a hair sample from pigs, we present the procedure used in the study by Wiechers et al. (2021) in which the authors evaluated the effect of housing in different types of farrowing pens in sows. Using an electric shaver, a bilaterally symmetrical area of 20 × 30 cm in the neck and shoulder blade region was shaved. This area was chosen to reduce the risk of contamination of the hair from the environment, such as with urine or faeces. It is also crucial to consistently take a hair sample from the same area of the body in all the individuals because the speed of hair growth and the concentration of cortisol in different areas of the body can vary (Heimburge et al. 2019). When collecting a hair sample, it is important to ensure that the sample contains actively growing hair. This can be achieved by using the so-called shave-reshave method (Meyer and Novak 2012) in which, to obtain samples of newly grown hair, this area was shaved twice during the experiment. The first shave took place 13 days after transferring the sows to the farrowing pen, ensuring that the sampled hairs included those that had grown since the relocation. The second shave was performed 13 days after the sows left the farrowing pen to include the entire period that the sows spent in the farrowing pen. The reason for such a timeframe is the fact that the depth of the hair shaft under the skin is 3 mm to 4 mm, and considering the rate of hair growth, which is 0.7 cm per month, it takes about two weeks for the lowest part of the hair shaft to reach the surface of the skin (Bacci et al. 2014). Hair samples are very stable and can be stored at room temperature for several months if protected from moisture and UV radiation (Heimburge et al. 2019; Heimburge 2021).

Laboratory processing consists of the extraction of cortisol and its determination. Prior to the actual cortisol extraction from the hairs, it is necessary to wash them to decontaminate them from external pollution (Pollock et al. 2021). Most of the published studies used isopropanol for hair washing, following the method described by Davenport et al. (2006). An organic solvent is used to extract cortisol from the keratin matrix of the hair. Before that, it is recommended to cut the hairs into short fragments or grind the sample into a powder. The cortisol extraction involves mixing the sample with methanol and incubating it. Incubation takes place overnight or up to 24 h at temperatures ranging from room temperature to 50 °C (Davenport et al. 2006; Cook 2012).

For the actual determination of the cortisol concentration, various studies used different analytical techniques, such as enzyme-linked immunosorbent assay, radioimmunoassay, enzyme-linked immunoassay (EIA), and HPLC-MS (Gow et al. 2010).

### Cortisol

Recently, there has been growing interest in the hair cortisol determination as a method that allows one to evaluate the long-term activation of the HPA axis, especially in livestock species (Heimburge et al. 2019; Heimburge 2021). Hairs accumulate cortisol over a longer period of time, enabling the assessment of stress levels and welfare of the pigs in the previous few weeks to months (Bacci et al. 2014; Everding 2021).

Using this matrix has several significant advantages. It is a method that is minimally invasive for the animals and is less stressful. The collection itself does not affect the concentration of cortisol in the collected sample (Creutzinger et al. 2017), is simple, and can even be performed by technical personnel (Everding 2021). Another advantage is that the concentration of cortisol in the hair is not subject to daily fluctuations in concentration.

Hair growth occurs in hair follicles in cycles. Active growth occurs only during the anagen phase, which is also the longest period and lasts for 3 to 5 months in pigs (Mowafy and Cassens 1976). The anagen phase is followed by a transitional phase called the catagen phase that lasts for around 20 days in pigs (Mowafy and Cassens 1976). The last phase is the telogen phase, when hair growth stops completely and is finally separated from the skin (Heimburge 2021). Due to domestication, there is no longer a clear distinction between spring and winter in terms of moulting. However, even in domesticated pigs, it was found that there is an increase in follicular activity at the beginning of summer, and the coat becomes denser (Mowafy and Cassens 1976; Heimburge 2021).

In mammals, cortisol is primarily transported in the blood by binding to proteins, with corticosteroid-binding globulin being the most significant. About 90% of systemic cortisol is bound to these proteins, with the remaining 10% existing unbound in the blood (Spencer and Deak 2017). This free, unbound cortisol can diffuse into the tissues, and cortisol concentrations detected in saliva are indicative of this free cortisol fraction (Bozovic et al. 2013; Heimburge 2021). The produced cortisol is then converted into biologically inactive and water-soluble metabolites, which are excreted in the urine and, to a lesser extent, in the faeces. Therefore, cortisol metabolites can also be determined in the urine and faeces (Palme et al. 1996).

The mechanism by which cortisol is incorporated into hair follicles has not yet been fully explained. Currently, four possibilities are under consideration. The first possibility appears to be the most important, suggesting that cortisol reaches the hair follicles through passive diffusion from the blood. This occurs during the growth phase of these follicles (Meyer and Novak 2012). The second possibility is that cortisol released from the sebaceous glands reaches the hair shaft (Mesarcova et al. 2017). The third possibility involves external contamination of the skin with material containing cortisol, which is then incorporated into the hair shaft (Heimburge et al. 2019). Fourthly, there is an independent ‘peripheral HPA-like system’ in the skin that produces cortisol, which then reaches the hair. This system is found in hair follicles, epidermal melanocytes, and dermal fibroblasts (Gow et al. 2010). According to some authors, cortisol production in this local skin system is considered negligible (Heimburge 2021). The exact mechanism has not been completely and reliably explained yet.

In addition to the effect of stress on the cortisol concentrations in pig hair, other factors have been identified that may be involved in the variability of its concentration in hairs, thus influencing the results. It is recognised that different results may be caused by different sampling methodologies, the area of the sample collection, and the analytical methods (Heimburge et al. 2019). For example, cortisol concentrations have been found to be lower in the craniodorsal regions of the pig body than in the dorsolumbar region (Casal et al. 2017).

Another factor influencing the cortisol concentration in pig hair may be the age. It was found that higher cortisol concentrations are present in young pig categories. Between the second and tenth weeks of life, piglets experience a significant decrease in the cortisol concentration in the hair. Compared to young animals, higher cortisol concentrations are found in the hair of sows (Heimburge et al. 2019; Heimburge et al. 2020).

A significant factor influencing the concentration of cortisol in pig hair is the hair colour. Dark hairs have been found to contain higher concentrations of cortisol compared to white hairs (Heimburge et al. 2019; Heimburge et al. 2020). The cause of this phenomenon has not yet been reliably explained, but a possible explanation may be that the increased amount of melanocytes facilitates the incorporation of lipophilic substances (Pragst and Balikova 2006). Furthermore, it is also possible that the melanin in dark hair may have a filtering effect on UV radiation and therefore inhibit the degradation of cortisol (Heimburge et al. 2020).

The amount of cortisol detected can also be affected by the analysed hair segment. It was found that the concentrations in the distal segments were significantly higher compared to the proximal segments (Heimburge et al. 2019; Heimburge et al. 2020). This can be explained by the fact that the distal hair segments are exposed to external influences for a longer time, making their surface more prone to damage. Therefore, the damaged part becomes more sensitive to external contamination with substances that contain cortisol, such as sebum, saliva, urine and faeces (Heimburge et al. 2020; Otten et al. 2020). This effect can be reduced by using the shave-reshave collection method, obtaining newly grown hairs and thereby minimising the possibility of external contamination (Meyer and Novak 2012; Heimburge et al. 2019).

The influence of the season also comes into consideration. A study by Wester et al. (2016) shows that sunlight and UV radiation degrade cortisol in hair. However, a study conducted in pigs (Heimburge et al. 2020) showed no effect of the season on the cortisol concentration in hair samples collected in summer and winter. It is important to note that the pigs were kept inside all the time, and thus, were not exposed to external influences.

In suckling piglets, the effect of chronic stress on the cortisol concentrations in the hair was determined after overcrowding and frequent mixing with unfamiliar piglets (Prims et al. 2019). After three weeks of exposure to stress, significantly higher concentrations of cortisol were found in the piglets’ hair compared to the control group (87.29 vs 75.60 pg/mg hair). The salivary cortisol concentrations were not significantly different between groups. This shows the better usability of the hair cortisol determination for the assessment of chronic stress. In another study in suckling piglets, Morgan et al. (2019) found that the hair cortisol levels from birth to weaning tended to be lower when invasive procedures were avoided, and environmental enrichment was provided.

The measurement of the cortisol concentration in the hairs of older pigs was also used to evaluate the possibility of reducing chronic stress by rearing pigs in an enriched environment. For example, the authors Casal et al. (2017) reported a significant reduction in the hair cortisol concentration in pigs provided with enrichment material for two months compared to pigs raised in barren environments. After two months, the hair cortisol was significantly lower for pigs raised in the enriched environment compared with the pigs raised in the barren conditions (3.72 ± 0.52 vs 7.58 ± 0.84 pg/mg hair, respectively). A combination of materials (sawdust, natural hemp ropes and rubber balls) was used to enrich the environment. In contrast, Nannoni et al. (2019) found no significant changes in the hair cortisol concentration in two independent trials with pigs testing different environmental enrichments (metal chain and wooden logs versus a hanging chain and an edible vegetal block).

Another application of cortisol determination in pig hair was for the pre-slaughter monitoring of pigs. Pigs with tail lesions throughout their lives were found to have higher hair cortisol concentrations compared to pigs with no tail lesions. Pigs in which movement disorders (lameness) were detected during their lives tended to have higher hair cortisol levels than non-lame pigs (lame: 52.72 ± 3.83 pg/mg, not lame: 43.07 ± 2.69 pg/mg) (Carroll et al. 2018).

Another area of application for the cortisol determination in pig hair involves sows. The housing of sows in single loose-housing systems in which animals were free to move was compared with conventional farrowing pens. The authors, Wiechers et al. (2021), found that the mean hair cortisol concentrations did not differ significantly between the systems (LH: 1.85 ± 0.82 pg/mg, FC: 2.13 ± 1.53 pg/mg).

## PIG WELFARE BIOMARKERS IN URINE AND FAECES

### Methodology of sampling, processing and analysis of samples

The stability of the measured hormones and their metabolites in faecal samples post-defecation is an issue due to rapid bacterial decomposition. Urine and faecal samples must be collected immediately and subsequently preserved, ideally, the samples must be frozen at –20 °C until analysis. Methods of co-preservation include freezing, freeze-drying, heat-drying, silica-drying, and storage in ethanol, formalin, acetic acid, and sodium hydroxide. For example, in a study by Wolf et al. (2020), the steroid concentration in faecal samples varied by 13% throughout a 48-h period; however, the glucocorticoid metabolites were relatively stable for approximately two days. Thus, the storage conditions are probably dependent on the marker that we want to analyse and the applied method.

The urinary concentration is a function of the adrenocorticotropic output and depends on the hydration level of the individual. A common method of correcting for the effects of dilution is to express the urinary cortisol as a ratio to the urinary creatinine. The most common method for determining catecholamines and corticosteroids in urine and faecal samples is HPLC-MS (Cook 2012); however, ELISA can be applied as well (Sciascia and Metges 2023).

Furthermore, it is necessary to mention that we must consider the metabolism of the analysed markers, too. For example, in the case of cortisol, approximately 93% of circulating cortisol is excreted in the urine and only the remaining 7% in the faeces after approximately 48 hours. Considering the level of microbial contamination and the presence of other metabolites of the organism, as well as the need to take samples at the right time interval and in the right way, it is necessary to consider whether the analysis of faecal and urine samples is suitable for the determination of welfare level markers in pig farms in the particular case. Likewise, the study by Mohan et al. (2020) points to the fact that the analysis of cortisol in saliva and blood samples is positively correlated, in contrast, the determination in urine is positively correlated with blood samples, but, at the same time, depends on the time. However, in the case of the determination of cortisol in faecal samples, the correlation with the analysis of blood is very weak.

### Cortisol

Contrary to saliva and blood samples, which give the results at the time of collection (acute stress reaction), measuring the concentration of cortisol in urine and faeces provides data about the longer-term effects of stress on pigs.

Corticosteroids in faeces are a complex of native hormones and metabolic products resulting from conjugation in the liver. The concentration of cortisol and its metabolites depends on the passage time of the digestive tract. The distribution of cortisol in the faeces is not homogeneous, and therefore, it is important to collect the entire faecal sample which can be considered as one of the sampling limitations. If there is a longer passage through the digestive tract, an increased concentration of glucocorticoids in the faeces is more likely to occur (Cook 2012). Mostl et al. (1999) found that after incubating the faeces at room temperature, 11,17-dioxoandrostane (a metabolite of cortisol) was detected in the faeces of domestic livestock, probably due to the bacteria present in the faeces, which can be considered as another limitation of this measurement.

The concentration of cortisol in the urine is a valid biomarker of stress, because the concentration in the urine is directly proportional to the free hormone in the blood. This is due to corticosteroids being bound to proteins and excreted by the kidneys. However, it is important to collect and analyse urine repeatedly over a 24-hour period to accurately diagnose the cortisol concentration. Data on the hydration level of a given individual are also important for measuring cortisol (Cook 2012).

Some studies considered the use of cortisol in urine and faecal samples as a biomarker of the stress load in pigs. For example, Faucitano et al. (2006) measured the urinary concentrations of cortisol in pigs at the slaughterhouse. Cortisol levels were affected by the fasting duration prior to slaughter; an increased concentration was noted in pigs fasted for 14 h before slaughter compared to pigs fasted for 4 h before slaughter. Francoise et al. (2002) measured urinary cortisol in pregnant sows housed in individual pens and collective pens, and no differences were found in the cortisol concentration between sows housed in individual and collective pens. Hay et al. (2003) evaluated the effect of castration on piglets by analysing behavioural changes and the concentrations of corticosteroids (cortisol, cortisone) and catecholamines (norepinephrine, epinephrine) in the urine. The behavioural signs showed that the castrated piglets were stressed, unlike the non-castrated ones. In this study, there was only an increased concentration of cortisone in the urine of the castrated piglets compared to the non-castrated piglets.

### Catecholamines

Catecholamines are synthesised in the brain, the adrenal medulla and sympathetic nerve fibres, and urine is the main elimination route of catecholamines from the body (Gurwitz and Ray 2024). The excretion products in urine sum up over several hours; it can therefore be considered a better method for determining the catecholamines in the urine than in the blood (Hay et al. 2000; Hay et al. 2001; Li et al. 2023).

Studies describe the measurements of catecholamines, i.e., epinephrine and norepinephrine, in urine as possible biomarkers of stress in animals. For example, Hay et al. (2001) measured urinary glucocorticoids (cortisol, cortisone), catecholamines (norepinephrine, epinephrine), and creatinine in piglets weaned 6 days postpartum compared to piglets weaned 28 days postpartum. In the piglets weaned earlier, an increased level of urinary cortisol and norepinephrine was observed on the first day following weaning. No changes in the urinary cortisol concentration were observed in piglets that were weaned up to 28 days. This increase was probably associated with emotional distress or acute food deprivation after early weaning.

Hay et al. (2000) monitored the HPA axis activity in pregnant sows and measured the cortisol and catecholamine levels in the blood and urine. Their results confirmed that the levels of the observed parameters correlated in the plasma and urine and that the values of catecholamines and corticosteroids were affected by the light/dark period. During the dark period, the concentrations of cortisol, cortisone, norepinephrine, and epinephrine were much lower than during the light period. At the same time, there was an increase in the urinary cortisol with the increasing stage of gestation, but there were no changes in the catecholamines with the stage of gestation.

In a study conducted by Wolf et al. (2020), faecal glucocorticoid metabolites (fGCMs) and cortisol levels showed an increase in pigs subjected to the ACTH stimulating test and after transportation. In pigs after ACTH administration, there was an overall fGCM increase of 180% above the baseline, while the cortisol increased by 110% compared to the baseline. The peak values occurred within 12 h to 24 h after the ACTH injection. There was an overall increase of 70% in the fGCM observed between 20 h to 48 h after the transportation of pigs lasting for 20 minutes.

Considering catecholamines and their analysis in urine samples, another possibility to use their potential to analyse the level of welfare in pig farms could be to determine the concentration of their metabolite, i.e., vanillylmandelic acid (VMA). The measurement of VMA can be conducted by the ELISA method or HPLC and is used in the screening of tumours (Tohmola et al. 2015). However, we did not find its use in determining the welfare level of pigs in any available study.

## CONCLUSION

The review elucidates the potential of a non-invasive approach to assess the stress levels and overall welfare in pig farms. As an alternative to invasive techniques, notably blood and plasma sampling, which serve as suitable biological matrices for determining these parameters, this review introduces information on a non-invasive approach to sampling. It suggests the exploration of potential biomarkers from the saliva, urine, faeces and hair of animals, providing insights not only into the established biomarkers from literary sources, but also addressing aspects of sample collection, storage and routine laboratory analyses. However, it should be noted that non-invasive techniques may not always be suitable, as they can be influenced by various parameters, as discussed within this review.
